# Temporary Carotid Occlusion Using a Hybrid Microsurgical-Endovascular Approach for Repair of Ophthalmic Artery Aneurysm

**DOI:** 10.7759/cureus.92957

**Published:** 2025-09-22

**Authors:** Daniel Jaraki, Amber Dorn, Thomas Eckert, Eesha Gurav, Cade Fallow, Nelson Gonzalez, Corey Collins, Roham Moftakhar

**Affiliations:** 1 Neurosurgery, University of South Carolina School of Medicine Columbia, Columbia, USA; 2 Neurosurgery, Prisma Health-Midlands, Columbia, USA

**Keywords:** endovascular surgical repair, hybrid surgery, microsurgical repair, neurosurgery, neurovascular disease, ophthalmic artery aneurysm, vascular surgery

## Abstract

A patient in their 50s with a history of anxiety, depression, bipolar disorder, and insomnia presented with two to three weeks of progressive altered mental status, including delusions and hallucinations. Emergency Department workup included non-contrast CT, which disclosed a mass along the anterior midline of the sella. Contrast MRI and computed tomography angiography (CTA) demonstrated a large (17 mm) saccular aneurysm at the para‑ophthalmic segment of the right internal carotid artery, adjacent to the ophthalmic artery origin. In a single hybrid session, the team first performed endovascular balloon‑assisted proximal carotid occlusion, then converted to an external approach via a right frontotemporal craniotomy to clip the aneurysm neck under direct visualization. The patient tolerated the combined endovascular‑microsurgical procedure without intraoperative complications. Postoperatively, they experienced a single seizure on night one but recovered fully after antiepileptic adjustment. Visual acuity improved from 2/200 to 20/100, and the patient was discharged home in stable condition.

## Introduction

Microsurgical interventions internal carotid artery

Ophthalmic artery (OA) aneurysms are the most common type of paraclinoid aneurysm and often result in visual impairments, headaches, and subdural hematomas [1,2]. These aneurysms can be occluded through open surgery, microclipping, or through coiled embolisms transported via endovascular microcatheters. Open microsurgical clipping can achieve immediate sac decompression but carries a higher rate of new post-treatment visual deficits compared with endovascular coiling in some series, whereas endovascular approaches avoid direct optic-nerve manipulation but may leave mass effect untreated or leave a higher rate of incomplete occlusion [1-3].

Microsurgical interventions for OA aneurysms include an anterior clinoidectomy (removal of the anterior clinoid process to decompress the optic nerve and expose the paraclinoid internal carotid artery (ICA)), optic strut removal, and distal dural ring dissection (the dural sleeve around the intradural ICA just distal to the clinoid); despite a notable shift toward an endovascular approach in recent years, the microsurgical approach is more favorable than the endovascular approach in terms of visibility of the aneurysm and immediate surrounding structures [3,4].

Endovascular surgical interventions

Endovascular techniques for treating OA aneurysms utilize a microcatheter placed through the femoral artery to occlude the aneurysm through detached coil embolism [5]. Stents on an additional microcatheter are often used to divert blood flow and navigate the detached coils [5]. There is also precedent for using balloon guides to navigate and establish proximal control (temporary reduction or cessation of antegrade flow in the parent artery, here achieved endovascularly with an inflatable balloon guide) during surgical treatment of ICA aneurysms [6]. An innovative “double-balloon trapping method” has been utilized for the treatment of complicated wide-necked aneurysms in small arteries. This technique involves the placement of proximal and distal balloons to reduce bleeding from the ruptured aneurysm, the rapid insertion of dense coiling in the aneurysm sac, and the deflation and removal of the balloons within five minutes of initiating this procedure. The overall advantage of this technique is its general effectiveness in treating complex, wide-necked aneurysms. For example, while preventing bleeding of the aneurysm, the placement of the two balloons prevents the coils from slipping back into the parent artery, all while stabilizing the coiling across the aneurysm. Although this offers a solution to a complex challenge, a few disadvantages of the double balloon technique should also be considered, including substantial risk for thromboembolic events during the procedure and prolapse of the mass of multiple coils following deflation of the balloons. These two risks could easily be addressed with regular, long-term angiographic follow-up, making this technique worth considering in cases of wide-necked aneurysms in small arteries [7]. The main advantage of using endovascular surgical interventions rather than the open approach in general is the capability to treat OAs without invasively opening the skull. However, in relatively small arteries (<3 mm), risk of intraoperative rupture, difficulty in aneurysm catheterization, and increased difficulty in placing coils illustrate some disadvantages of the endovascular technique. Use of soft coils and balloon assistance helps to reduce these risks [8]. In one study of 166 carotid-ophthalmic aneurysm treatments, 26 out of 147 treated with an endovascular approach required further intervention, while six out of 23 microsurgeries resulted in major complications. This result indicates that invasive microsurgeries have a higher risk of adverse effects, but endovascular coil embolizations have a greater rate of recurrence and incomplete occlusion [9,10].

Existing evidence of a hybrid open-endovascular approach

Hybrid neurosurgical approaches combine open and endovascular techniques to treat complex conditions such as aneurysms, arteriovenous malformations (AVMs), skull base tumors, and dural arteriovenous fistulas (AVFs) [11]. Recent studies show benefits including rapid aneurysm securing with clot evacuation or thrombectomy, single-session AVM embolization with resection, and tumor devascularization before removal, all with favorable outcomes [11]. These strategies improve safety and efficacy in cases where one modality alone may be insufficient [11]. In the treatment of multiple cerebral aneurysms, including a right OA aneurysm, hybrid treatment was used to embolize the OA before clipping two remaining left-sided aneurysms. Despite the complexities of the case, the patient recovered within six months without any neurological deficits [12]. A proximal balloon guide was also utilized to gain proximal control of a vertebrobasilar junction aneurysm through the right vertebral artery, before the aneurysm was clipped. This hybrid approach was considered to be a safe measure for treatment, and the operation proceeded successfully [13].

In this case, we present the treatment of an OA aneurysm using an endovascular balloon guide and microsurgical clipping. We want to contribute to the growing body of evidence for the use of hybrid neurosurgical techniques to increase the efficacy of certain aneurysm treatments. This case demonstrates an intraoperative, real-time conversion from endovascular proximal control to open microsurgical clipping to achieve decompression while preserving ophthalmic-artery perfusion, followed by staged flow diversion to treat a small remnant.

## Case presentation

Presentation/preoperative course

A patient in their 50s presented with a past medical history of anxiety, depression, bipolar disorder, and insomnia to the emergency department with altered mental status. Per interviewing and collateral information from family, the altered mental state began two to three weeks prior to presentation and acutely worsened over the previous two days. Per the patient’s daughter, the patient was acting strange and delusional, walking around barefoot and claiming strangers were offering the patient drugs and stealing from the patient. On arrival, the patient expressed some suicidal ideation but did not plan to act on the ideation and had no history of suicidal attempts in the past. In addition, the patient reported auditory and visual hallucinations as well as being equipped with special powers, but was unable to elaborate. Patient reported drinking three to four beers per day and reported marijuana use, denying use of cocaine, methamphetamines, heroin, or other illicit substances. Patient denied any chest pain, dyspnea or labored breathing, abdominal pain, nausea, vomiting, diarrhea, constipation, headache, visual changes, fever, or chills.

**Figure 1:**
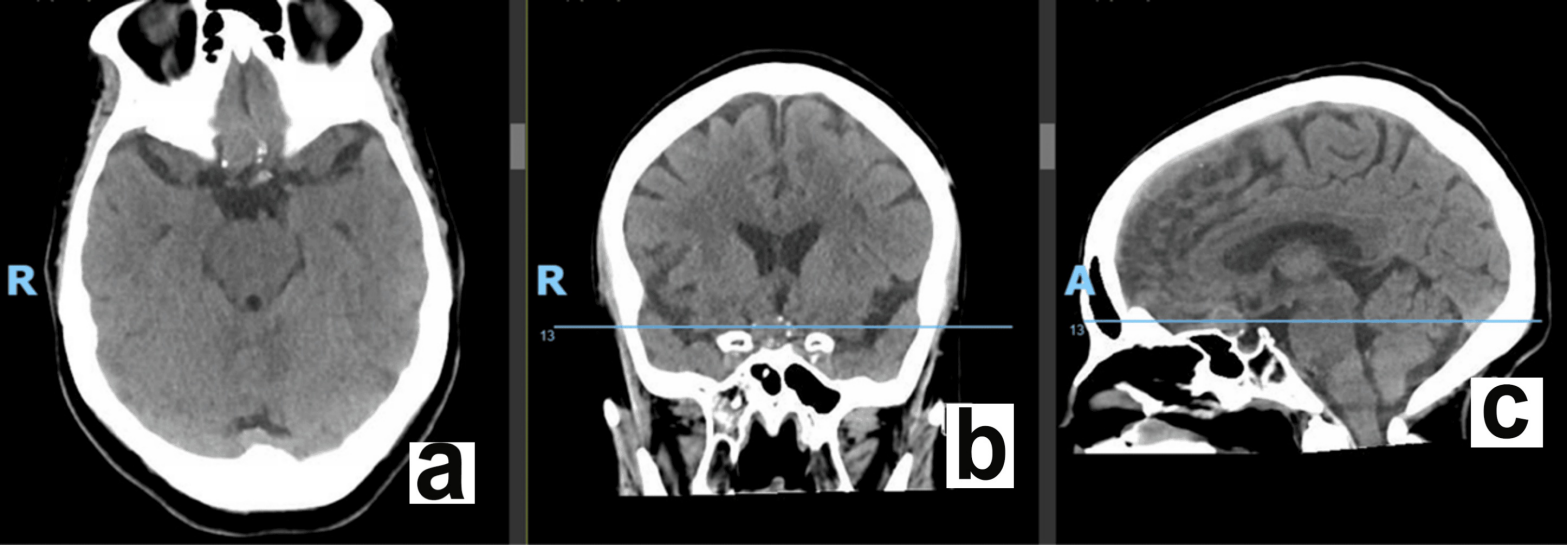
Preoperative axial (a), coronal (b), and sagittal (c) head CT scans. Masslike abnormality just anterior to the sella near midline measuring 1.4 x 1.0 x 1.4 cm.

A noncontrast head CT in the patient’s workup demonstrated no intracranial hemorrhage but did show a mass-like abnormality with peripheral calcification along the anterior edge, along the sella near the midline, measuring approximately 1.4 x 1.0 x 1.4 cm (Figure 1).

**Figure 2:**
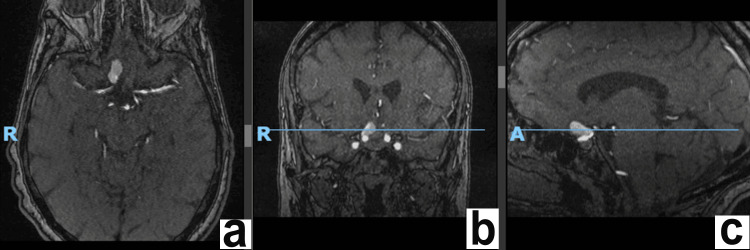
Preoperative axial (a), coronal (b), and sagittal (c) cuts of MRI angiogram. Demonstrating a large right paraophthalmic segment ICA aneurysm (measuring up to 17 mm), which arises at or just distal to the ophthalmic artery takeoff. This projects anterosuperiorly along the planum sphenoidale. ICA: internal carotid artery.

Based on appearance, diagnostic considerations included aneurysm or partially calcified meningioma, and MRI/MRA was suggested for further initial evaluation. Neurosurgery was consulted, and the patient was ultimately admitted with an MRI angiogram and CT angiography of the head and neck pending. The patient was ultimately found to have a large, saccular aneurysm arising off the paraopthalmic segment of the ICA, which demonstrated a neck measuring approximately 5 x 3 mm and a neck to dome height of approximately 10 mm. A maximum sac diameter of 17 mm with a small amount of likely mural aneurysm thrombus along the left aspect of the aneurysm sac was noted (Figure 2).

Description of hybrid procedure

The patient, with an unruptured right ophthalmic artery aneurysm with right visual field deficit, was taken to the operating room for microsurgical clipping with temporary occlusion of the cervical carotid artery using an endovascular method.

The patient was placed supine on the table in the hybrid operating theater and underwent successful induction of general anesthesia and endotracheal intubation. The groin areas were prepped and draped in sterile fashion. Then, using the Seldinger technique, the right common femoral artery was accessed using an 8 French by 10-cm sheath. The carotid arch was navigated using a flowgate balloon guide 8 French catheter along with a large 5 French Berenstein. The right common carotid artery was selectively catheterized. A right cervical carotid angiogram AP lateral projection was performed, which demonstrated no significant stenosis at the bifurcation. Next, the right ICA was selectively catheterized, and the 8 French flowgate balloon guide was placed in the right ICA. At this point, under fluoroscopy, the balloon guide was inflated with 0.2 cc, and the test occluded the right ICA by injecting contrast and assessing for flow. After confirmation, the balloon was deflated and the guide was removed.

The patient’s head was placed in a Mayfield clamp with all pressure points well-padded, and the Stealth navigation system (Medtronic, Minneapolis, MN, USA) was successfully registered. An incision starting from the zygoma was marked in a curvilinear fashion and made using a 10-blade, reflecting the skin anteriorly. The pericranium was harvested, followed by a temporalis muscle dissection and reflected inferiorly. A right frontotemporal craniotomy was performed, and the rest of the surgery was completed under direct vision of the operative microscope.

The left wing of the sphenoid was drilled, and the skull base was entered by performing a cranial orbital approach by exposing the lateral wall of the cavernous sinus. Cranial nerve V2 was identified, and its dura propria was removed. The superior orbital fissure was unlocked, allowing for the identification of the anterior clinoid and optic canal. A full anterior clinoidectomy was performed, allowing for decompression of the optic nerve and roof of the optic canal. At this point, the carotid artery was able to be identified extradurally in the paraclinoid segment. Using direct vision under the operative microscope, the dura was opened and retracted along the Sylvian fissure to the optic nerve. The optic nerve was decompressed by opening up the falciform ligament and the optic dura. Next, the distal ring around the carotid artery was partially resected, releasing the distal dural ring. Returning to the Sylvian fissure and its dissection, the M1, M2, carotid artery, oculomotor nerve, anterior choroidal artery, and the posterior communicating artery were identified. Here, the neck of the aneurysm was identified; the distal ophthalmic artery aneurysm was dissected, and then the proximal neck of the ophthalmic artery was identified.

Under direct vision with fluoroscopy, the carotid arch was navigated, and a balloon guide was placed in the right ICA. The patient was given 3500 units of IV heparin, and the activated clotting time (ACT) was checked five minutes later, which demonstrated above 220. The balloon guide was inflated, and the right ICA was proximally occluded. Returning to the head, the aneurysm was noted to be much softer after applying suction to the guide catheter. A 10mm temporary clip was placed in the supraclinoid ICA for trapping of the aneurysm. Once the neck was identified, a fenestrated clip was applied to the neck and top of the aneurysm. The distal clip off of the supraclinoid ICA was taken off, the balloon guide was deflated, and an intraoperative angiogram using indocyanine green was performed as the origin of the ophthalmic artery was noted to be coming off of the carotid next to the neck of the aneurysm and was occluded. The angiogram demonstrated that the aneurysm was not filling, but the ophthalmic artery was not filling either, with the supraclinoid ICA having adequate flow. At this point, the balloon guide was inflated again, and the distal clip at the supraclinoid ICA was put back on, removed, and two more fenestrated clips were applied for occlusion. A dogear was noted, and two more clips were placed. Here, another intraoperative angiogram using ICG was performed after taking off the distal clip and deflating the balloon guide. This angiogram demonstrated adequate flow through the ophthalmic artery and supraclinoid ICA, with the aneurysm showing no filling. After clipping the aneurysm, the guide catheter was removed from the ICA, and the 8 French area was closed using a closure device. Hemostasis was obtained, and a piece of muscle was placed in the clinoidal space. A dural substitute was placed on top of the bone, and using a KLS Martin system (KLS Martin Manufacturing LLC, Jacksonville, FL, USA), a subgaleal drain was placed and closed using 2-0 Vicryl suture (Ethicon, San Angelo, TX, USA). The temporalis was closed using 2-0 Vicryl as well, and the skin was closed with staples. The patient tolerated the procedure well with no intraoperative complications.

Post-operative course 

The patient was extubated postoperatively and was able to move all extremities against gravity; however, the patient did not follow commands nor open their eyes. The first night post-op, the patient began seizing and vomited, but this resolved before medications were given, and the patient was subsequently intubated for airway protection, and the dosage of levetiracetam was adjusted. After the seizure, the patient's exam was similar, but now the patient was able to follow commands. The patient was put on long-term monitoring (LTM) electroencephalography (EEG) after this episode, but no seizures were captured while it was in use.

The following day, an MRI brain without contrast was obtained, which was negative for a large territorial infarct, showing a thin postoperative subdural collection with minimal mass effect. The patient was then extubated the next day to high-flow nasal cannulae and was later weaned to room air. Physical Therapy/Occupational Therapy (PT/OT) was consulted and recommended rehab. The patient was discharged with home health and instructed to follow up with neurosurgery in two weeks.

Three weeks after discharge, the patient presented to their primary care provider (PCP) reporting falling and hitting the back of their head two days prior, after restarting their zolpidem. The patient denied headache but stated increased sensation of confusion compared to baseline, which the patient attributed to zolpidem use. No focal neurologic weaknesses on physical exam were present, and the patient was counseled by PCP to obtain a non-contrast CT head, but the patient declined. One week later, the patient presented to the ED complaining of right-sided frontal headache but denied vision changes, tingling, or weakness of any extremities. Neurologic exam at this time was wholly normal. Neurosurgery was consulted, and CTA head and neck showed residual filling of a right ophthalmic artery aneurysm measuring up to 6 mm. The patient was then discharged and scheduled for a diagnostic cerebral angiogram in a month, which showed a residual aneurysm of approximately 5 mm at the neck of the clipped aneurysm. At this time patient endorsed continued loss of vision in the right eye.

The following month, the patient reported for pipeline stent embolization of the residual right ophthalmic artery aneurysm. After the procedure, the patient was admitted to the ICU and noted to be drowsy but otherwise without abnormal exam findings except for swelling of the L groin at the angiogram site. A CTA was obtained significant for a left common femoral artery (CFA) bifurcation pseudoaneurysm extending into the anterior groin with a large surrounding hematoma.

At the bedside, a femoral stop was placed by neurosurgery. It was reported by the patient the following day that vision in the right eye had improved from blackness to blurry vision. Later that day, ultrasound-guided treatment of the left pseudoaneurysm with thrombin was performed by interventional radiology. Vascular surgery was consulted; however, repeated femoral ultrasounds demonstrated no evidence of pseudoaneurysm or arteriovenous fistula,and the vascular service was subsequently discharged from the case. Several days later, the patient was discharged home.

The patient returned to the ED the following week with a chief complaint of bleeding from a femoral hematoma, but was seen by vascular surgery and deemed stable to return home, given ultrasound showed no flow in the previous pseudoaneurysm, and there was no active bleeding at that time. Two months later, the patient presented to the ED for headaches on the right side going into the back of their neck, which had been occurring for three weeks. A CTA head and neck was obtained, which showed ​​a small 2 mm outpouching near the base of the treated right ophthalmic artery aneurysm, which was mildly decreased in size when compared to five months prior, in addition to sequelae of multifocal intracranial clipping and no evidence of new intracranial arterial abnormality. The patient was then discharged home.

## Discussion

Ophthalmic artery aneurysms at the paraclinoid segment of the internal carotid artery present significant challenges due to their proximity to the optic nerve and the complex anatomy of the skull base. In this case, combining endovascular balloon occlusion for proximal control with microsurgical clipping allowed for precise aneurysm sac decompression and clip placement under direct vision. Despite meticulous technique, a small OA aneurysm persisted, necessitating staged pipeline stent embolization.

The patient’s transient postoperative seizure and delayed pseudoaneurysm at the femoral access site highlight the importance of comprehensive perioperative management, including seizure prophylaxis and close vascular monitoring. Ultimately, the patient’s visual function improved, and no further aneurysm growth was observed on follow-up imaging, indicating that the hybrid strategy can offer durable control in complex cases.

Endovascular coiling minimizes direct optic nerve handling but may not promptly relieve mass effect in broad-neck paraclinoid aneurysms [14]. These observations are supported by a comparison study that noted 70% of patients with preoperative visual impairment recovered vision after balloon-assisted clipping, suggesting that controlled proximal flow reduction and targeted decompression at the neck can reverse compressive optic neuropathy when collateral flow is preserved [14]. Zhang et al. reported that post-treatment visual deficits were significantly more common after microsurgical clipping than after coiling (16.1% vs. 2.4%, p=0.010) [15], likely reflecting greater risk of optic apparatus manipulation and perforator compromise during open surgery [1,15]. Our case illustrates how a hybrid workflow of proximal balloon control with microsurgical decompression and precise clip placement, followed by staged flow diversion for a small remnant, can leverage the visual safety profile of endovascular therapy while achieving the mechanical decompression benefits of surgery. Such an approach can not only avoid new visual injury but also facilitate recovery of preexisting deficits [14,15].

As a single patient case report, our findings are inherently limited by the absence of a control cohort and the potential for selection bias. Furthermore, the reliance on a hybrid operating suite and specialized endovascular expertise may limit the generalizability of this approach to centers without comparable resources.

## Conclusions

This case highlights the successful application of a hybrid microsurgical-endovascular approach for treating a complex ophthalmic artery (OA) aneurysm, demonstrating how this technique merges the precision of microsurgery with the minimal invasiveness of endovascular methods. By employing endovascular balloon-assisted proximal control alongside microsurgical clipping, we achieved near-complete occlusion with a small remnant treated with staged flow diversion while mitigating risks to the optic nerve and reducing the likelihood of recurrence. The patient’s visual outcome improved from a best-corrected visual acuity of OD 2/200 pre-procedure to OD 20/100 in the immediate postoperative period, and recovery further underscores the safety and efficacy of this strategy.

Because this is a single-case report, conclusions about comparative efficacy or safety are necessarily limited; however, the case suggests that hybrid proximal balloon control can enable direct sac decompression under vision while preserving ophthalmic-artery perfusion and that staged endovascular flow diversion can address small residuals. Larger, controlled series with standardized neuro-ophthalmic follow-up are required to define the reproducibility, safety profile, and long-term visual and angiographic outcomes of this hybrid paradigm.

## References

[1] Kamide T, Tabani H, Safaee MM, Burkhardt JK, Lawton MT (2018). Microsurgical clipping of ophthalmic artery aneurysms: surgical results and visual outcomes with 208 aneurysms. J Neurosurg.

[2] Heran NS, Song JK, Kupersmith MJ, Niimi Y, Namba K, Langer DJ, Berenstein A (2007). Large ophthalmic segment aneurysms with anterior optic pathway compression: assessment of anatomical and visual outcomes after endosaccular coil therapy. J Neurosurg.

[3] Michalinos A, Zogana S, Kotsiomitis E, Mazarakis A, Troupis T (2015). Anatomy of the ophthalmic artery: a review concerning its modern surgical and clinical applications. Anat Res Int.

[4] Gross BA, Du R (2013). Microsurgical treatment of ophthalmic segment aneurysms. J Clin Neurosci.

[5] Ban SP, Kwon OK, Lee SU (2018). Long-term outcomes of patients with stent tips embedded into internal carotid artery branches during aneurysm coiling. AJNR Am J Neuroradiol.

[6] Ohshima T, Kawaguchi R, Matsuo N, Miyachi S (2021). Local balloon-assisted navigation of a microcatheter into an aneurysm during intracranial aneurysmal coiling: a dunk shot technique. Asian J Neurosurg.

[7] Ansari A, Ohshima T, Goto S, Yamamoto T, Ishikawa K, Kato Y (2019). Double-balloon trapping for coil embolization of ruptured internal carotid artery aneurysm: a novel technique. Asian J Neurosurg.

[8] Zang P, Liang C, Shi Q (2010). Endovascular embolization of very small cerebral aneurysms. Neurol India.

[9] Yadla S, Campbell PG, Grobelny B, Jallo J, Gonzalez LF, Rosenwasser RH, Jabbour PM (2011). Open and endovascular treatment of unruptured carotid-ophthalmic aneurysms: clinical and radiographic outcomes. Neurosurgery.

[10] Gu W, Zhou G, Aldiyarova A (2023). Stent-assisted coiling of intracranial carotid ophthalmic segment aneurysm segment aneurysms: long-term follow-up from a single center. J Interv Med.

[11] Ulmer S, Gruber P, Schubert GA, Remonda L, Marbacher S, Grüter BE (2024). Combined microsurgical and endovascular intracranial aneurysm treatment: interdisciplinary experience using a true hybrid approach and a systematic review of the literature. Brain Sci.

[12] Rotim K, Kalousek V, Splavski B, Tomasović S, Rotim A (2021). Hybrid microsurgical and endovascular approach in the treatment of multiple cerebral aneurysms: an illustrative case series in correlation with literature data. Acta Clin Croat.

[13] De Vilalta À, López P, Sanmillán JL, De Miquel MÀ, Barranco R, Gabarrós A (2022). Endovascular assisted vertebrobasilar junction aneurysm clipping in a hybrid operation room. Case report. Brain Spine.

[14] Lu G, Chung J, Park JC, Ahn JS, Kwun BD, Lee DH (2022). Comparison of visual outcomes of ophthalmic artery aneurysms treated with microsurgical clipping and endovascular coiling. Neurointervention.

[15] Zhang T, Cai Y, Wang L (2023). Visualization balloon occlusion-assisted technique in the treatment of large or giant paraclinoid aneurysms: A study of 17 cases series. Front Neurol.

